# Commentary: Selpercatinib versus multi-kinase inhibitors for advanced medullary thyroid cancer: a network meta-analysis of RET-targeted therapies

**DOI:** 10.3389/fendo.2026.1979615

**Published:** 2026-10-05

**Authors:** Arnau Domenech, Martí Hopmans, Erin Barker, Joe W. E. Moss, Montserrat Carrasco

**Affiliations:** 1 Esteve Pharmaceuticals S.A., Barcelona, Spain; 2 York Health Economics Consortium, University of York, York, United Kingdom

**Keywords:** advanced medullary thyroid cancer, commentary, multi-kinase inhibitors, network meta analysis (NMA), RET-targeted therapies

We have read with great interest the published network meta-analysis (NMA) by Wang et al. (2026) in Frontiers in Endocrinology, which synthesised available randomised controlled trial (RCT) evidence to evaluate the relative efficacy and safety of rearranged during transfection (RET)-targeted agents for the treatment of advanced medullary thyroid cancer (MTC) (1). Through a comprehensive systematic literature review and NMA, their study provides relative effect estimates comparing RET-targeted agents, addressing a critical evidence gap due to the lack of direct evidence. Five studies were included in the NMA: ZETA (vandetanib) (2), EXAM (cabozantinib) (3), ALTER01031 (anlotinib) (4), LIBRETTO-531 (selpercatinib) (5), and EORTC-1209 (nintedanib) (6). All studies used placebo as the control arm except LIBRETTO-531, which used physician’s choice of cabozantinib or vandetanib. It should be noted that the publication incorrectly references the phase 1–2 LIBRETTO-001 trial instead of the phase 3 LIBRETTO-531 trial (reference 13 in Section 2.6.2 of the original publication). While the study demonstrated that selpercatinib exhibits superior efficacy, in terms of progression-free survival (PFS) and objective response rate (ORR), and a more favourable safety profile compared with other RET-targeted agents for advanced MTC, there are some important points for consideration that may affect the interpretation of the results. However, it should be noted that we did not reperform the NMA as part of this commentary, and the points we raise are methodological rather than quantifying their impact on the derived treatment effect estimates.

Firstly, in four of the five studies included, eligible patients were required to have documented disease progression. However, this criterion was not required in the ZETA trial (2). Differences in the median PFS in the placebo arms across trials, derived from observed Kaplan-Meier plots, suggest that patients included in the ZETA trial were healthier than patients enrolled in other trials where disease progression was an eligibility criterion (**Table 1**). If disease progression is a treatment effect modifier, this will introduce bias and violate the assumption of transitivity in the NMA. Therefore, it could be argued that the patients from the ZETA trial included in the current analysis represent a different treatment population compared to the other studies included in the analysis. However, it should be noted that despite these potential differences in population, the NMA-derived PFS treatment effect for cabozantinib versus vandetanib was not statistically significantly different (HR 0.61; 95% CI 0.35 to 1.05).

**Table 1:** Median progression-free survival (PFS) time for the placebo arm across studies.

Study (NCT number)	n	Median PFS (months)	PFS definition
ZETA (NCT00410761)	100	19.3	Investigator assessment
EXAM (NCT00704730)	111	4.0	Blinded IRC
ALTER01031 (NCT02586350)	29	11.1	IRC
EORTC-1209 (NCT01788982)	19	2.9	Investigator assessment

IRC, Independent review committee; NCT, National Clinical Trial number; PFS, progression-free survival.

The LIBRETTO-531 trial is not included since it did not include a placebo arm.

An alternative approach would be to include the results of subgroup analyses from the ZETA trial, where in a progressive and symptomatic subgroup, the median PFS was 8.40 months in the placebo arm (7). Results from the progressive and symptomatic subgroup are more likely to align with the patient population of other trials and remove a source of potential bias in the current analysis; though, we acknowledge that this breaks randomisation. However, this approach was taken in a previous NMA conducted during an independent Health Technology Assessment (HTA) which addressed the potential differences in the EXAM and ZETA populations by using the ZETA progressive and symptomatic subgroup (8). This analysis also found no statistically significant difference in PFS between vandetanib and cabozantinib (HR 1.14; 95% CI 0.41 to 3.09).

Due to the absence of a placebo arm in the LIBRETTO-531 trial, the authors adopt a weighted anchoring strategy based on the observed treatment distribution in the LIBRETTO-531 control group. The authors derived a synthetic “physician’s choice” control arm by taking a weighted average of the log-transformed effect estimates from the EXAM (cabozantinib vs. placebo) and ZETA (vandetanib vs. placebo) trials. This allowed selpercatinib to be linked to the central placebo node of the network. The weights used were proportional to the proportion of drug usage LIBRETTO-531 (74.2% cabozantinib and 25.8% vandetanib) and variance was propagated into the network using the delta method. It is unclear whether a two-stage approach was taken whereby the estimated relative effects of selpercatinib vs. placebo were calculated outside of the NMA model and then reintroduced into the network. If a two-stage approach was used, it would raise two important concerns.

1. Given that the EXAM trial and the ZETA trial are included in the network, a two-stage approach could result in double counting and could fail to account for the correlation introduced by reusing the EXAM and ZETA estimates.
2. Although the delta method can propagate the sampling variance of the component estimates, its validity depends on whether all relevant covariance terms and other sources of uncertainty were incorporated.

The confidence intervals observed for selpercatinib vs. placebo estimates are narrower than expected, given that the estimated relative effects rely on data from the EXAM trial and the ZETA trial. Furthermore, the correlation potentially introduced by reusing the EXAM and ZETA estimates may not have been captured and may lead to violations of variance consistency.

Additionally, for PFS, standard NMA using hazard ratios assumes that there is a constant treatment effect over time. The authors provided no evaluation (e.g., log-log survival plots or inspection of reconstructed Kaplan-Meier curves) to confirm whether the proportional hazard (PH) assumption holds. If the PH assumption is violated, single summary HRs are mathematically invalid, and time-varying NMA models (such as Fractional Polynomials) should have been used instead. Therefore, if the PH assumption is potentially violated in this study, the transitivity assumption may not hold, and treatment ranking may be distorted.

Finally, although none of the pairwise differences for PFS or ORR were statistically significant, the numerical direction of the ORR comparison favoured vandetanib, whereas the PFS comparison numerically favoured cabozantinib. This inconsistency in the available published evidence, together with the wide confidence intervals, illustrates the limitations of interpreting treatment rankings as evidence of comparative superiority. Therefore, this further demonstrates why direct evidence is required to compare the efficacy of vandetanib to cabozantinib.

In summary, this study provides a comparative analysis of RET-targeted agents for advanced MTC. To strengthen the findings, we suggest that the authors consider subgroup results for the ZETA trial to account for differences in documented progression status between studies and perform the necessary checks to ensure the PH assumption holds for PFS. Most importantly, to ensure reliable and methodologically robust results, a single-stage, joint likelihood framework should be adopted to include the LIBRETTO-531 trial in the network, due to the lack of a placebo arm if a two-stage approach was used by the authors. It should be noted that as part of this commentary, we did not reperform the NMA and therefore, we cannot quantify the exact impact that correcting the identified issues would have on the derived treatment effect estimates beyond those currently presented that have previously been conducted by other research groups.
